# A Large Intramural Lipoma Causing Asymptomatic Colocolic Intussusception in an Adult

**DOI:** 10.1155/cris/9950558

**Published:** 2025-06-02

**Authors:** L. Keiber, B. Geissler, M. Anthuber

**Affiliations:** Universitätsklinikum Augsburg, Augsburg, Germany

## Abstract

**Background:** Intestinal intussusception is a common condition in children, and the cause is often idiopathic. In contrast, adult intussusception is rather rare and almost always secondary due to an underlying condition such as a tumor, inflammatory disease, or a diverticulum. Hence, the treatment almost always is surgical resection of the lesion.

**Methods:** We retrospectively analyzed a case of asymptomatic intussusception in a male adult using patient data retrieved from the hospital patient database. This includes findings from both physical and radiological and endoscopical examinations. The patient was contacted 4 weeks and 6 months postsurgery for a clinical follow-up.

**Aim:** Until this day, there is no guideline regarding the underlying pathology. Hence, this case report wants to contribute to a field where there is only insufficient patient data.

**Results and Discussion:** We presented a case of colocolic intussusception in an adult caused by a large intramural lipoma. After a full gastrointestinal diagnostic protocol and interdisciplinary case discussion, we decided to offer surgical resection, from which the patient recovered quickly. The benign nature of the tumor and the complete lack of symptoms despite significant tumor size make this case particularly interesting. We emphasize the need for a larger study group to create robust data that aid in creating care guidelines.

## 1. Introduction

Intussusception is a surgical condition in which a proximal part of the intestine folds into an immediately adjacent distal bowel segment. In children younger than 3 years, it is the most common cause of intestinal obstruction, and clinical presentation often involves abdominal pain, palpable abdominal mass, and bloody stools. In these cases, the origin is typically idiopathic, and therapeutic hydrostatic enemas are often sufficient to achieve intestinal repositioning [1–3].

In contrast, intussusception is a rather uncommon cause for bowel obstruction in adults and almost always has an identifiable cause. Here, folding of the intestine into itself is often facilitated by bowel propulsion, where an intestinal segment is pushed toward a distinct lead point (i.e., tumor and diverticulum) or mechanical lesion (i.e., intraabdominal adhesion). A 2017 study analyzed intussusception etiology for different age groups [4]: They found that in adults, the majority of cases with a distinct lead point pathology are caused by neoplasms, at least half of which are malignant. With 75%–80% of cases occurring in the small intestine, the colocolic intussusception is rather rare and, unlike in the small intestine, mostly caused by malignant lesions such as colorectal adenocarcinoma [5]. The most common benign nonepithelial-derived cause in the large intestine is a colonic lipoma [6].

Here, we present a case of colocolic intussusception caused by an intramural lipoma in a male Caucasian patient in his 60s. Despite the lipoma being rather large (4.0 cm), he presented with unspecific symptoms, making this case particularly interesting.

## 2. Case Presentation

A 63-year-old male patient reported to his general practitioner (GP) with more than 4 weeks of diffuse abdominal pain, originating in the lower left abdomen. In the previous year, the patient was diagnosed with prostate cancer and underwent minimally invasive radical prostatectomy. Other than that, there is no medical or surgical history, and the patient did not experience weight loss or irregular bowel movements. He had no family history regarding bowel pathologies.

**Figure 1:**
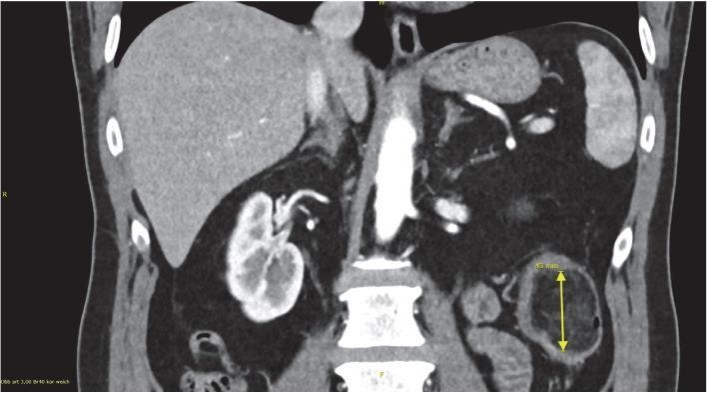
Coronary CT slice showing the intramural lipoma near the left colic flexure. Diameter shown by yellow line (~43 mm). CT scans are particularly sensitive for lipomas larger than 2 cm. The lesion matches the absorption density of −80 to −120 Hounsfield units that is described in other case reports and contemporary radiological references for intestinal lipoma [7, 8].

**Figure 2:**
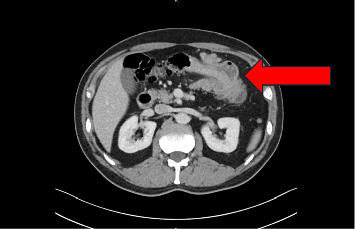
Axial CT showing colocolic intussusception in the left hemiabdomen.

**Figure 3:**
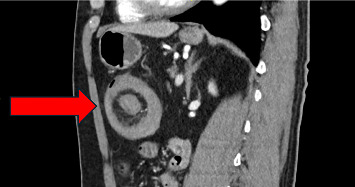
Sagittal CT of intussusception, displaying the characteristic “target sign” or “donut” morphology.

The GP suspected acute diverticulitis of the descending colon and started our patient on lactulose. This, however, did not result in a significant improvement in symptoms. After a few days, the patient presented to the emergency department of a local primary care hospital with a singular event of rectal bleeding during defecation. Here, he was started on amoxicillin/clavulanic acid and was dismissed the same day. Subsequently, due to persistence of abdominal symptoms, the GP ordered an abdominal computed tomography (CT) scan, which suggested a case of large bowel intussusception adjacent to a fatty tissue mass in the vicinity of the left colic flexure (Figures 1–3). The patient was then referred to our hospital for evaluation of treatment options.

Upon arrival in our emergency department, the patient presented diffuse lower abdominal pain. There was no sign of peritonism, vitals were within normal range, and digital rectal examination did not reveal pathological findings. Laboratory investigation demonstrated a white blood cell (WBC) count of 12.7/nL, an elevated CRP of 15.5 mg/dL, and otherwise unremarkable results.

Upon closer inspection of the CT scan by our in-house radiology department, uncomplicated diverticulitis of the sigmoid was reported additionally (classified as stage 1b).

We decided to admit the patient to our department of general surgery for intravenous antibiotic therapy and to evaluate surgical options regarding the intussusception. Within 5 days of treatment with ampicillin/sulbactam, symptoms decreased completely and both CRP and WBC declined significantly. Here, we decided against explorative surgery and discharged the patient temporarily in a good general state of health.

However, at that time, the benign or malignant nature of the tumor remained unclear. Therefore, 2 weeks later, we performed a diagnostic colonoscopy, where the tumor's macroscopic impression did not propose malignancy. Histological analysis of biopsies taken from the lesion were inconclusive but suggested an adipocyte-derived benign neoplasia.

We discussed possible treatment options with the patient. Because of tumor size, patient age, and inability to rule out malignancy, we recommended partial colectomy as an elective procedure within 2 weeks, to which the patient agreed. Tumor markers CEA and CA19-9 were within normal range in the patient's blood.

The day prior to surgery, the tumor was marked by endoscopy-assisted injection of ink ~70 cm from anal verge. As mentioned above, the tumor appeared to be near the left colic flexure in the CT scan. When we performed diagnostic laparoscopy, however, the lesion was mostly in the proximal transverse colon. Hence, we performed open partial transverse colectomy with a primary anastomosis, removing ~15 cm of bowel.

**Figure 4:**
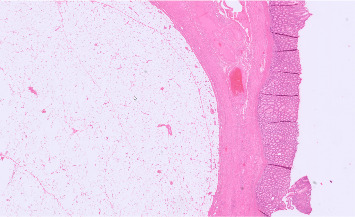
Histological examination of the lipoma, which is located in the submucosa layer of the large intestine. Healthy intestinal glands are depicted in the right half of the picture; mature adipocyte tissue can be seen to the left.

Upon closer inspection of the removed sample, a rather large and soft mass was palpable. Histologic examination showed mature adipocytes within the tumor. No signs of malignancy were reported; MDM2 stain was negative. These findings are consistent with an intramural lipoma, measuring 4.0 cm × 3.5 cm × 3.5 cm (Figure 4).

The patient's postoperative recovery was uneventful, and he was discharged on day 6 after surgery. He was clinically examined in our outpatient clinic within a month after surgery, where he was symptom-free. Within another 9 months after surgery, no episodes of abdominal pain were reported.

## 3. Discussion

Colonic lipoma is a rare benign mesenchymal tumor of the large intestine which has first been described in the eighteenth century [9]. A series of several thousand autopsies analyzed by Weinberg and Feldman [10] in 1955 reports an incidence of 0.2% for lipomas of the gastrointestinal tract. More recent data suggest a colonic lipoma incidence between 0.2% and 4.4% [6, 11]. A contemporary review by Menegon Tasselli et al. [12] describes the transverse colon as the most frequent location (28% of cases) for colonic lipomas causing intussusception. In contrast, other studies report that more than 90% of cases are located in the right side of the large bowel. The most abundant histological origin appears to be the submucosa [13, 14]. Affected patients can present with various unspecific symptoms such as anemia and abdominal pain. However, according to M'Rabet et al. [15], it is highly unlikely that a lesion of the size reported here does not clinically manifest. Although several studies have assessed whether there is a gender predominance, there are contradicting results regarding this matter. However, there is broad agreement that the diagnosis is predominantly found in patients between 40 and 70 years of age [11, 16]. Hence, we can conclude that our index patient fits into the expected age range and his lesion matches previously reported anatomical landmarks.

Colocolic intussusception is rather rare in adults and presents a significant diagnostic challenge due to nonspecific symptoms [17–19]. Most studies describe that the likelihood of occurrence of symptoms increases with lipoma size, with lesions larger than 2.0 cm being highly likely to cause clinical manifestations. Typical symptoms include rectal bleeding, abnormal bowel movements, and abdominal pain [5, 13]. Although some of those were reported by our patient, it is more plausible that his symptoms were a clinical manifestation of diverticulitis. Therefore, the case reported here is rather unusual: We incidentally detected a 4.0 cm large colonic lipoma causing asymptomatic intussusception.

Current literature indicates that preoperative diagnostic methods for intussusception in adults should include a CT scan as a cornerstone to further clarify the etiology. Lipomas smaller than 2.0 cm, however, are difficult to detect in diagnostic imaging [7, 8]. Colonoscopy is sometimes recommended for unclear lesions. However, the risk of bowel perforation must be weighed against possible benefits, as histological results can be inconclusive as our case demonstrates [13, 20].

Small asymptomatic lesions can be either observed or removed endoscopically. Especially pedunculated colonic lipomas are good candidates for ablation by therapeutic colonoscopy [21]. Our department of gastroenterology assessed the possibility of endoscopical resection for our index patient. However, given the anatomy and the size of the lesion, this did not appear to be an adequate option.

Currently, there is no guideline that outlines a coherent standard of care for the pathology presented in this case report. Hence, treatment plans for patients with large intestinal lipoma have to be set up individually. There is a broad consensus on the need of surgical therapy of intussusception in adults, especially when certain risk factors are met. These include symptomatic intussusception, lead point intussusception in CT imaging, and colocolic processes [4, 22]. Additionally, in the category of lead point pathologies, tumor size can be seen as a risk factor on its own due to the high risk of underlying malignancy in larger processes [23].

Therefore, in accordance with current literature, we decided to offer our patient elective partial colectomy, from which he recovered without long-term effects on his general health.

## 4. Conclusion

As mentioned above, intussusception is a rare condition in adults and can pose a diagnostic and therapeutic challenge to any physician. We presented a case of asymptomatic intussusception caused by a large lipoma serving as a lead point.

This clinical report showcases that surgeons must be familiar with the etiology and diagnosis of adult intussusception to provide adequate treatment for affected patients, as surgical intervention is the mainstay of treatment in these cases.

As of publication of this paper, there appears to be a lack of up-to-date cohesive and systematic reviews regarding intussusception in adults. Most current publications on the topic are either outdated or focused on specific subtypes of the disease. On top of that, the literature appears to be inconsistent regarding epidemiology and clinical presentation as we laid out in the previous section. Hence, with this report, we want to contribute to a field where only insufficient data exist. We suggest that further cases similar to ours have to be collected in order to create coherent and robust data regarding the underlying pathology. This could help in meeting the demand for a comprehensive study based on a broader dataset and, subsequently, aid in deriving adequate treatment options.

## Data Availability

The data that support the findings of this study are available on request from the corresponding author (L. Keiber). The data are not publicly available due to contents that could compromise the privacy of the patient reported here.
